# Fluctuating neurological symptoms: should I call the neurologist or the hematologist?

**DOI:** 10.1515/almed-2020-0082

**Published:** 2020-11-12

**Authors:** Rita Losa-Rodríguez, Carmen Pérez Martínez, Gabriel Rodríguez Pérez, Ignacio de la Fuente Graciani, Lara M. Gómez García

**Affiliations:** Service of Laboratory Analysis, Hospital Clínico Universitario de Valladolid, Valladolid, Spain; Servicio de Hematología y Hemoterapia, Hospital Clínico Universitario de Valladolid, Valladolid, Spain

**Keywords:** schistocytes, thrombocytopenia, thrombotic microangiopathy

## Abstract

**Objectives:**

The objective of this study was to highlight the role of the clinical laboratory and the relevance of reporting the case immediately to the unit of hematology for the diagnosis and early administration of treatment in the presence of such an urgent hematologic disease as thrombotic thrombocytopenic purpura (TTP).

**Case presentation:**

An elderly patient was referred to the emergency department of our hospital by his general practitioner for speech difficulty, facial asymmetry and weakness in the upper limb. Stroke code was activated. However, laboratory findings (anemia, thrombocytopenia, elevated creatinine, total bilirubin and LDH, negative direct Coombs test) and presence of schistocytes in the peripheral blood smear test were consistent with a completely different diagnosis: TTP thrombotic microangiopathy.

**Conclusions:**

The first diagnostic approach of left hemispheric stroke was not confirmed in the laboratory, with findings of nonautoimmune hemolytic anemia, thrombocytopenia without apparent cause and presence of schistocytes. We should not forget that the clinical manifestations of this condition are widely variable and may include multiorganic dysfunction. Although confirmation of diagnosis is based on ADAMTS-13, its associated high mortalitiy requires immediate treatment on mere suspicion.

## Introduction

Thrombotic thrombocytopenic purpura (TTP) is a life-threatening, acute hematologic process characterized by microangiopathic hemolytic anemia and thrombocytopenia. Its clinical manifestations are the result of its physiopathology: nonautoimmune hemolytic anemia, severe thrombocytopenia, fluctuating neurological manifestations, renal dysfunction and fever, although the two latter are less frequent [1], [ 2].

The disease can be congenital or acquired as a result of a deficiency or dysfunction of protein disintegrin and metalloproteinase with a thrombospondin type 1 motif, member 13 (ADAMTS13). This protease mediates the breakdown of large multimers of von Willebrand factor (vWF) into smaller units. Large vWFs bind to platelets, which results in blood clots that occlude small vessels and cause ischemic endothelial damage. This cascade primarily affects cerebral and renal microcirculation. This originates severe thrombocytopenia for platelet consumption, hemorrhagic anemia, microangiopathic anemia and organ damage [1], [ 3].

Diagnostic suspicion of thrombotic microangiopathy (TAM) is based on the presence of hemolytic anemia secondary to red blood cell fragmentation (schistocyte) and thrombocytopenia without an apparent cause, along with the presence of signs and symptoms of organic damage. Upon diagnosis of TAM, consider TTP as the first possible entity and immediately start plasma exchange and steroid therapy until ADAMTS13 test results confirm diagnosis of TTP [4].

## Case presentation

An 86-year-old man was referred by his primary care to the emergency department (ED) who presented with speech problems, several-minute facial drop and right arm weakness that did not resolve completely. In the ED, the patient had another episode with the same symptoms. Stroke code was activated, and the patient was examined by the on-call neurologist.

Findings of examination included the presence of petechiae in the limbs and chest, conjunctival jaundice and mild neurologic symptoms (3 points on the National Health Institute Scale).

Laboratory analysis revealed significant bicytopenia (hemoglobin 81 g/L [120–180 g/L] and a platelet count of 6 × 10^3^/μL [150–400 × 10^3^/μL]) confirmed by peripheral blood smear (PBS), with schistocytes not visualized. It showed altered biochemistry (creatinine 1.54 mg/dL [0.7–1.2 mg/dL], total bilirubin 2.53 mg/dL [0.10–1.20 mg/dL] at the expense of indirect bilirubin, LDH 1110 UI/L [135–250 UI/L]) and negative direct Coombs test results, and absence of ionic alterations or hepatic dysfunction.

CT scan of the brain did not demonstrate early signs of ischemia and hemorrhage. CT angiography did not show extracranial or intracranial occlusion, and no alterations were observed on cerebral perfusion maps. However, fluctuating symptoms of the left hemispheral stroke persisted. In view of the analytical findings, the start of antiplatelet therapy was postponed, and the patient was left in observation in the stroke service. In this clinical setting, TAM was suggested as first diagnosis after cross-consultation with the unit of hematology.

**Figure 1:**
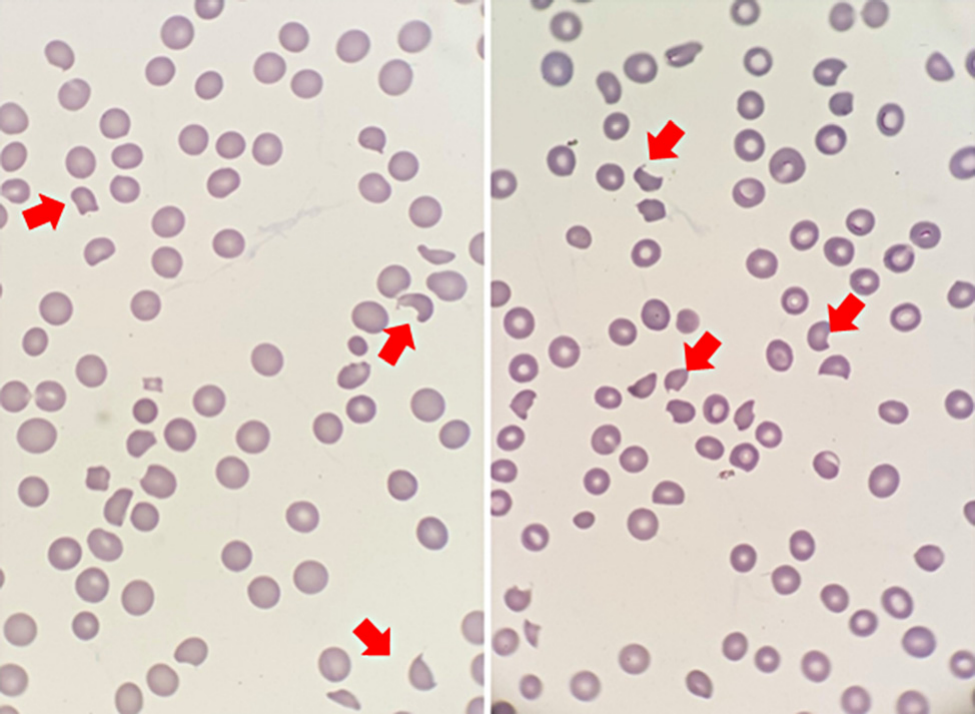
Peripheral blood smear at diagnosis. Anisopoikilocytosis with presence of schistocytes and confirmed thrombocytopenia. Red arrows indicate typical schistocyte morphology. May-Grunwald-Giemsa staining (×1000 optic microscopy).

A repeated laboratory analysis demonstrated severe anemia (hemoglobin 74 g/L), persistent severe thrombocytopenia (platelet count 13000/μL; without citrate alterations) and intense hemoglobinuria on the urine reactive strip test. This time, PBS revealed the presence of anisopoikilocytosis and schistocytes (Figure 1).

Clinical and analytical data are consistent with a TTP TAM after exclusion of other secondary causes of TAM.

Intensive corticosteroid therapy is started at a dose of 2 mg/kg/day, and a daily therapeutic plasma exchange (TPE) regimen was initiated after a blood sample was drawn for ADAMTS13 testing. ADAMTS13 activity was 0% (ADAMTS13 activity <5% is indicative of TTP) with the presence of ADAMTS13 inhibitor [5].

TPE was performed daily using fresh frozen plasma as a solution until day 6, with a progressive increase in platelet count that reached >150 × 10^3^/μL in 2 consecutive days. Then, TPE was performed at 48 h intervals, without the patient showing a decrease in platelet count.

The patient showed good tolerance to TPE and only developed paresthesias around the mouth and in the tips of the fingers and toes. These symptoms are secondary to consumption hypocalcemia induced by the use of citrate in TPE. After close analytical monitoring and calcium gluconate supplementation, symptoms disappeared.

**Figure 2:**
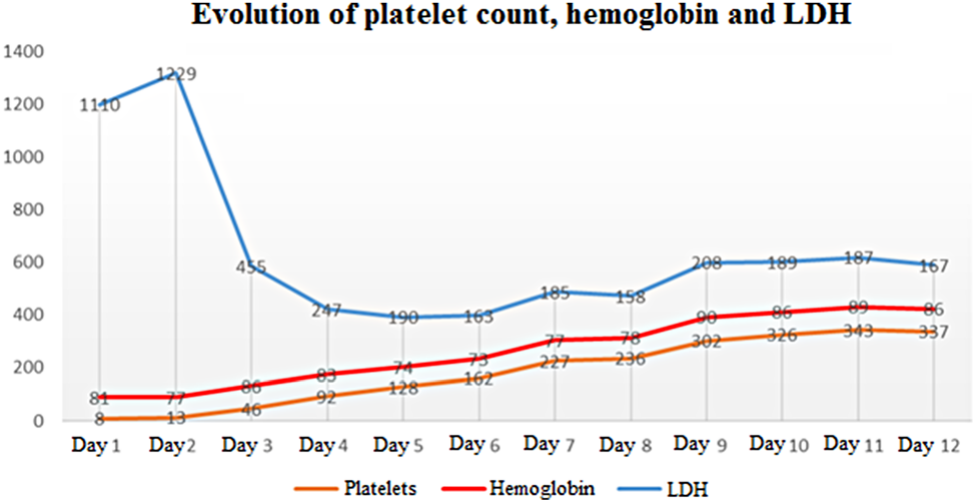
Evolution of the platelet count “×10^3^/μL”, hemoglobin “g/L” and LDH “UI/L” from diagnosis to therapeutic plasma exchange (TPE) completion.

The clinical course of the patient was favorable, with normalization of platelet count and anemia (Figure 2). Hemolysis was resolved, and the patient remained asymptomatic after eight sessions of TPE. Finally, the patient was discharged with follow-up in the outpatient unit. Corticosteroid therapy at a dose of 1 mg/kg/day was maintained and progressively reduced until suspension.

## Discussion

The incidence of TTP in the USA is 4–6 patients per 1 million/year. No accurate data are yet available in Spain, although a national group of TTP is developing a national registry. If left untreated, TTP causes death in 85–90% of patients, mainly due to ischemic events. However, a significant proportion of survivors have a poor quality of life, with depressive syndrome and neurocognitive deficiency [6], [7], [8].

TTP is idiopathic in more than 90% of cases, although there are reported cases of TTP caused by medication (ticlopidine, clopidogrel, tacrolimus), autoimmune diseases or pregnancy. In less than 5% of cases, TTP is congenital (Upshaw-Schulman syndrome) due to mutations in the ADAMTS13 gene, with it being more severe in children and adolescents [9].

Initial diagnosis of TTP is clinical, supported by laboratory data suggestive of the disease. The most frequent presentation is hemorrhagic diathesis (66%), followed by neurological abnormalities (66%) and fever (24%). The platelet count is relevant, as severe thrombocytopenia (platelet count <50 × 10^3^/μL) and presence of schistocytes in PBS are suggestive of TTP. Other signs of nonautoimmune hemolytic anemia are decreased hemoglobin, elevated reticulocytes, elevated LDH, hyperbilirubinemia with elevation of the indirect fraction, decreased haptoglobin and negative direct Coombs test results accompanied or not by a moderate increase in creatinine and hemoglobinuria [10], [11], [12].

PBS is of especial relevance in this entity since the presence of schistocytes is key to differential diagnosis. Schistocytes are caused by the mechanical fragmentation of RBCs in areas of turbulent blood flow surrounding microvascular thrombi. Schistocytes can be found in other clinical settings with similar rheologic conditions, as prosthetic valve or intravascular stent dysfunction, intravascular metastases of some adenocarcinomas, microangiopathy associated with hematopoietic stem cell transplantation, autoimmune diseases, infections, associated with drugs and so on; therefore, the presence of schistocytes is necessary but not sufficient to confirm diagnosis. However, a rate of 1 schistocyte in 100 RBCs is highly suggestive of TAM [13], [ 14].

Determination of ADAMTS13 activity confirms TTP diagnosis in the presence of an absolute deficit (<5% activity), both in the congenital and the acquired form of the disease. The presence of anti-ADAMTS13 antibodies is positive in the acquired form, whereas it is negative in the congenital form. However, as ADAMTS13 tests are not routinely performed in laboratories, they are sent to a laboratory of reference, with a turnover time of 24–48 h. In this context, the *PLASMIC score* emerges as a useful diagnostic tool. This algorithm uses a score system based on predictive variables of severe ADAMTS13 deficiency (<10% of activity), which classifies the risk of acquired TTP. These variables include platelet count, hemolysis data, mean corpuscular volume, INR, serum creatinine, history of cancer and hematopoietic or solid organ transplantation [15], [ 16].

Predictive factors of a poor prognosis include elevated LDH (>10 times above the upper normal limit), an advanced age and elevated ultrasensitive troponin T levels (>0.25 ng/dL). The latter must be monitored to assess myocardial damage in patients with a diagnosis of TTP in the asymptomatic stage [17].

TTP is a hematological emergency that requires urgent treatment. When other causes are excluded, TTP must be suspected in the presence of microangiopathic hemolytic anemia and thrombocytopenia, and treatment must be immediately started. The treatment of choice includes TPEs with plasma replacement, by which the patient’s anti-ADAMTS13 antibodies are eliminated, and deficient enzyme supplementation. Corticosteroid therapy is also needed, as immunosuppression inhibits anti-ADAMTS13 antibodies. A set of studies have demonstrated its effectiveness, with a reduction in mortality and the number of TPE needed [4], [ 18].

Other therapeutic options are biological therapies. Rituximab (an anti-CD20 agent, used in lymphoproliferative and autoimmune diseases) administered concomitantly to TPE is indicated in the early stages of the disease and in patients with high risk of mortality (neurological or cardiac involvement). This agent is also indicated in refractory and asymptomatic cases and is related to a lower rate of relapse and earlier response. The novel caplacizumab (inhibits vWF-platelet interaction) was recently approved by the AEMPS (Spanish Agency for Drugs and Health Products) and is recommended in combination with TPE and immunosuppression. This agent accelerates platelet count normalization and is associated with a lower incidence of mortality and TTP recurrence [10], [19], [20].

In the last years, advances in the understanding of the physiopathology of the disease have facilitated earlier diagnosis and immediate treatment. At present, the prognosis of the disease has improved and morbidity and mortality rates have decreased.
